# *QuickStats: *Percentage* of Residential Care Community^†^ Residents with a Fall,^§^ by Census Region — United States, 2016^¶^

**DOI:** 10.15585/mmwr.mm6737a6

**Published:** 2018-09-21

**Figure:**
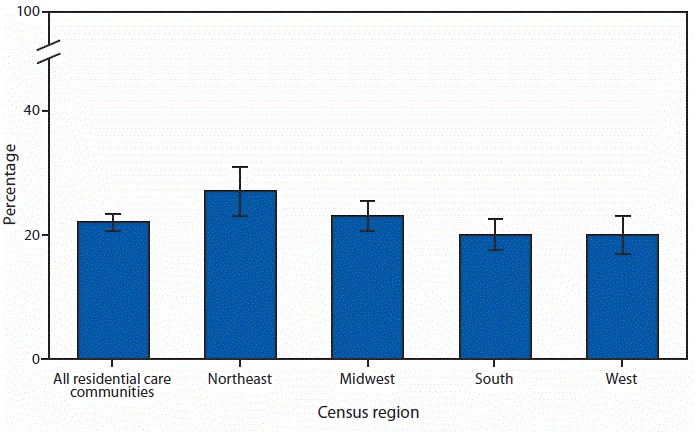
In 2016, 22% of current residents living in residential care communities had a fall in the past 90 days, representing 175,000 residents in the United States. By region, 27% of residents living in communities in the Northeast, 23% of residents in Midwest communities, and 20% of residents in communities in the South and West, respectively, had a fall. A higher percentage of residents in the Northeast had a fall compared with residents in the South and West.

**Source:** National Study of Long-Term Care Providers, 2016 data. https://www.cdc.gov/nchs/nsltcp/nsltcp_rdc.htm.

